# A risk prediction model of spontaneous miscarriage in women with threatened miscarriage: a prospective cohort study

**DOI:** 10.3389/fmed.2025.1669594

**Published:** 2025-11-11

**Authors:** Chee Wai Ku, Yu Bin Tan, Marie Min Tse Tan, Sze Ing Tan, Carissa Shi Tong Ng, Jyotsna Ramakrishna, Hiu Gwan Chan, Thiam Chye Tan, Jerry Kok Yen Chan, See Ling Loy

**Affiliations:** 1 Department of Reproductive Medicine, KK Women's and Children's Hospital, Singapore, Singapore; 2 Duke-NUS Medical School, Singapore, Singapore; 3 Maternal and Child Health Research Institute, KK Women's and Children's Hospital, Singapore, Singapore; 4 Yong Loo Lin School of Medicine, National University of Singapore, Singapore, Singapore; 5 Lee Kong Chian School of Medicine, Nanyang Technological University, Singapore, Singapore; 6 Paediatric Endocrinology Service, KK Women's and Children's Hospital, Singapore, Singapore; 7 Obstetrics and Gynaecology Academic Clinical Programme (OBGYN ACP), SingHealth Duke-NUS Academic Medical Centre, Singapore, Singapore; 8 Department of Obstetrics and Gynaecology, KK Women's and Children's Hospital, Singapore, Singapore

**Keywords:** threatened miscarriage, risk prediction model, miscarriage prediction, serum progesterone, clinical triage tool

## Abstract

**Introduction:**

This study aims to develop a holistic risk prediction triage tool for women with threatened miscarriage by integrating clinical, biochemical, and radiological factors. Additionally, we aim to assess the performance of various models incorporating different factor combinations, adaptable for diverse resource settings.

**Methods:**

This prospective cohort study included 1,080 women with single intrauterine pregnancies at 5–12 weeks' gestation presenting with threatened miscarriage at KK Women's and Children's Hospital, Singapore between October 2017 and May 2023. Multivariable logistic regression and risk-score models were developed using maternal age, nausea, prior miscarriages, serum progesterone, gestational age, and fetal heart activity. Model performance was assessed via Area Under Receiver Operating Characteristic (AUROC) curve with 10-fold cross-validation. The primary outcome was miscarriage by 16 weeks.

**Results:**

The miscarriage rate was 17.3%. Low serum progesterone (< 35 nmol/L) was the strongest predictor [odds ratio (OR) 26.3; 95% confidence interval (CI): 16.6–41.5], followed by absent fetal heart detection (OR 4.05; CI: 2.37–6.92) and increasing maternal age (OR 1.05; CI: 1.00–1.11). Higher gestational age decreased miscarriage risk (OR 0.57; CI: 0.45–0.73). The integrated model demonstrated high predictive accuracy (AUROC 0.90; CI: 0.87–0.93). The risk-score model achieved AUROC 0.82, with 80% sensitivity and 84% specificity at cut-off of 2.

**Discussion:**

This clinically practical holistic prediction tool enables early identification of women at high risk of miscarriage following threatened miscarriage. It may guide personalized counseling and targeted interventions in both high- and low-resource settings.

## 1 Introduction

Threatened miscarriage, defined as vaginal bleeding without cervical dilation in an early intrauterine pregnancy (1), is the most prevalent early obstetric complication, affecting up to 20% of all pregnancies (2). Approximately one in four patients progresses to spontaneous miscarriage (3), causing significant distress on affected women and their families (4), and incurs considerable economic costs to individuals, healthcare system and society-at-large (5). Despite these repercussions, effective screening and risk stratification algorithms to predict outcomes and personalize management remain lacking.

Since 2021, both the International Federation of Gynecology and Obstetrics and National Institute for Health and Care Excellence (NICE) recommend progesterone supplementation in women experiencing early pregnancy bleeding based on a prior history of miscarriage, a key triage criterion used to guide management (1). However, its inconsistent efficacy highlights the limitations of relying on a sole factor, underscoring the complexity and heterogeneity of miscarriage pathophysiology (6, 7). This highlights a critical need for more personalized and effective strategies that can accurately assess and mitigate miscarriage risk.

Miscarriage management is challenging due to its multifactorial etiology (8). Risk factors include maternal clinical, biochemical and radiological markers. Notable clinical risk factors are advanced maternal age (9), high body mass index (BMI) (10) and reduced nausea (11). Biochemically, low serum progesterone levels have also been associated with an increased risk of miscarriage (12–14). Progesterone is a vital hormone in early pregnancy, supporting endometrial receptivity, embryo implantation, and placental development. Even in the presence of ultrasound findings such as a gestational sac, serum progesterone provides complementary functional information on corpus luteal and early placental adequacy, thereby enhancing assessment of pregnancy viability (12, 15). Our group demonstrated that serum progesterone levels in early pregnancy are significantly lower in pregnancies complicated by threatened miscarriage compared to normal pregnancies (12). This finding paves the way for a serum progesterone screening tool to guide the management of patients presenting with threatened miscarriage (14). Radiologically, lower gestational age at time of presentation with threatened miscarriage and the absence of fetal heart (16) on ultrasound are significant predictors of spontaneous miscarriage. Despite advances in identifying individual risk factors, a comprehensive, integrative risk stratification tool remains lacking. Such a model could substantially improve personalized care and early intervention.

This study aims to develop a novel risk prediction triage tool for women presenting with threatened miscarriage by integrating clinical, biochemical, and radiological factors. We also evaluate different model combinations to support use across diverse healthcare settings.

## 2 Material and methods

This study was reviewed and approved by the SingHealth Centralized Institutional Review Board (CIRB) of Singapore (CIRB Reference no. 2017/2638). The Strengthening the Reporting of Observational Studies in Epidemiology (STROBE) reporting guideline was followed and written informed consent was obtained from all participants enrolled in the study. All research described in this manuscript adhered to the Declaration of Helsinki (17).

### 2.1 Study participants & procedures

This single-center cohort study utilized prospective data from women presenting with threatened miscarriage at the Urgent O&G Center (UOGC) of KK Women's and Children's Hospital (KKH) between 6th October 2017 and 20th May 2023, in Singapore. Women with a single confirmed intrauterine pregnancy between 5- and 12-weeks' gestation dated via transvaginal ultrasonography attending our UOGC for threatened miscarriage (per vaginal bleeding with an intrauterine gestational sac and fetal pole) were included. Exclusion criteria included women who received progestogens during the current pregnancy, those with multiple gestations, in-vitro fertilization (IVF) pregnancy, incomplete, missed (defined as a crown–rump length ≥7 mm in the absence of embryonic heart rate), or inevitable miscarriage, pregnancy of unknown location, ectopic pregnancy, termination of pregnancy and those without a fetal pole. At the initial visit, the attending clinician conducted a targeted history taking, physical examination, and ultrasound evaluation. Serum progesterone was measured as routine clinical practice. The history focused on questions related to the presenting complaint. Women with serum progesterone below 35 nmol/L were considered “high-risk” for miscarriage (18) and were treated with oral dydrogesterone according to the manufacturer's protocol (Duphaston, Abbott), provided anticipatory guidance, and monitored closely. Conversely, women with serum progesterone levels of 35 nmol/L or higher were classified as “low-risk” and managed conservatively with counseling and reassurance, without the use of progestogens. All study participants were reviewed in person in the outpatient clinic within 2 weeks of initial presentation with threatened miscarriage, including follow-up ultrasounds. They were subsequently monitored until 16 weeks' gestation to ascertain pregnancy outcome, via review of medical records. There was no follow-up visit or ultrasound scan at 16 weeks' gestation.

### 2.2 Assessment of exposure variables

Maternal age, ethnicity (Chinese, Malay, Indian, Others), number of previous miscarriages, presence of pain and nausea were recorded at the time of recruitment via an investigator-administered questionnaire in either English or Chinese. Maternal BMI was calculated using each patient's measured height (in cm) and weight (in kg) on the day of their visit. BMI was categorized as underweight, normal weight, overweight and obese (< 18.5, 18.5–24.9, 25–29.9, ≥30 kg/m^2^) according to the World Health Organization BMI criteria (19). Per-vaginal bleeding was quantified using a pictorial blood assessment chart, which rates the extent of staining on disposable sanitary products on a scale from 0 to 4 (20).

Maternal blood samples were collected at the recruitment visit in plain tubes and centrifuged for 10 min at 3,000g within 2 h of collection, and serum progesterone was subsequently measured in the KKH clinical laboratory using a commercial ARCHITECT progesterone kit (Abbott, Ireland).

Gestational age was determined via the crown-rump length by the attending physician (Registrar and above) using a transvaginal sonography device (Philip HS50) (21). The mean gestational sac diameter (MSD) was determined by averaging measurements from three orthogonal planes (in mm). Pregnancy failure is diagnosed if there is an absence of fetal pole when the MSD is ≥25 mm on ultrasound (16).

### 2.3 Assessment of pregnancy outcome

The outcome of interest was spontaneous miscarriage, defined as self-reported uterine evacuation following an inevitable or incomplete miscarriage, or complete miscarriage with an empty uterus by 16 weeks' gestation (16). Data were retrieved from medical records.

### 2.4 Statistical analyses

Baseline maternal demographics and pregnancy characteristics were statistically compared between women with ongoing pregnancies at 16 weeks' gestation and those who miscarried. Continuous variables were analyzed using a two-sample *t*-test and are presented as mean values with standard deviations. Categorical variables were analyzed using the Chi-square test and are presented as frequencies and percentages.

Multiple imputation was applied using chained equations with fully conditional specification (22). Univariable and multivariable logistic regression models with Firth correction were used to identify variables associated with the odds of miscarriage. Quantitative association from these models are reported as odds ratios (ORs) with corresponding 95% confidence intervals (CIs). Performance metrics for the prediction models were represented by area under the receiver operating characteristic (AUROC) curves scores, based on different combinations of pre-selected maternal characteristics. These included (a) clinical characteristics such as maternal age (9), absence of nausea (11), number of previous miscarriages; (b) serum progesterone levels (12, 13) and (c) ultrasonographic factors such as gestational age (16) and absence of fetal heart (16). The characteristics were selected based on literature review, usage of a direct acyclic graph, and corroboration with univariable analyses (*p* < 0.15) (23). The AUROC value of >0.80 indicates that the model exhibits a good discriminatory ability (24). The lowest Akaike information criterion (AIC) indicates the best fit based on existing data. Firstly, a full model with all predictors included was considered. Then, variables which were not statistically significant were removed from model 1, yielding model 2. Model 3 consisted of clinical and biochemical factors while model 4 consisted of clinical and radiological factors. Lastly, model 5 consisted of clinical factors only. The final predictive model of miscarriage was chosen to develop a risk score model based on the criteria of having the highest AUROC and the lowest AIC. No imputation was applied because there was < 0.05% of participants with missing data in this model (*n* = 5/1,080). Maternal age was categorized into below 40 and 40 years or older (25, 26). The optimal cut off point of gestational age was determined as 6.36 weeks in receiver operating curve (ROC) analysis using Youden index. Score values for each independent variable were assigned based on the range of β coefficients estimated from the multivariable logistic model. A score of 0 was assigned as the reference category of each variable. The total risk scores were then categorized into low risk (0–2), medium risk (3–5) and high risk (6–8) based on the distribution of miscarriage rates. The optimal cut-off point was selected at the threshold where both sensitivity and specificity exceeded 80% (27).

In addition, a 10-fold cross-validation was performed to assess the performance of the multivariable logistic regression models and risk-score predictive model (28). This cross-validation procedure helps to provide estimates of its predictive ability and generalization performance. The 95% CIs of AUROC were calculated using bootstrapping with 2,000 replications. Logistic regression was performed on the complete dataset (*n* = 881) as a sensitivity analysis to examine the robustness of the imputed models. Statistical significance was set at *p*-value < 0.05 and all tests were two-tailed. Statistical analyses were performed using SAS version 9.4 (SAS Institute Inc., Cary, NC), Stata 18 (Stata Corporation), R packages “mice” and “pROC”.

## 3 Results

### 3.1 Maternal characteristics

Among 1,938 women screened, 584 women met one or more exclusion criteria. We excluded women who had received progestogen during the current pregnancy (*n* = 2), multiple gestations (*n* = 6), pregnancy of unknown location (*n* = 3), ectopic pregnancy (*n* = 2), IVF pregnancy (*n* = 2), termination of pregnancy (*n* = 26) and those with no fetal pole present (*n* = 544) (Appendix 1). Of the 1,353 women enrolled, 13 women were excluded from data analysis as their gestational age determined by ultrasound was not between 5 and 12 weeks, 246 women were lost to follow-up and 12 had unknown fetal pole status. Of the 1,080 women eligible for analyses, 187 miscarried at 16 weeks' gestation and 893 women had an ongoing pregnancy. The overall miscarriage rate at 16 weeks' gestation was 17.3%.

Maternal demographic and clinical characteristics by miscarriage status at 16 weeks of gestation are summarized in Table 1. Women who had an eventual miscarriage were older (31.7 vs. 30.8 years), less likely to experience nausea (76.0 vs. 64.8%), and had low serum progesterone levels (< 35 nmol/L) (73.3 vs. 6.9%) and hence more likely to receive progesterone supplementation at the UOGC (72.7 vs. 13.5%), as compared to those with ongoing pregnancies at 16 weeks. Additionally, the fetus was more likely to be of a lower gestation age (6.20 vs. 7.51 weeks) and to have no fetal heart (47.6 vs. 6.9%) detected during the ultrasound evaluation.

**Table 1:** Baseline maternal characteristics by miscarriage status at 16 weeks' gestation.

	**All**	**Miscarriage status at 16 weeks' gestation**		***P*-value**
**Characteristic**	**(*****n*** = **1,080)**	**Ongoing pregnancy (*****n*** = **893)**	**Miscarriage (*****n*** = **187)**	
**Clinical factors**				
Maternal age, years (SD)	31.0 ± 4.44	30.8 ± 4.26	31.7 ± 5.17	0.043
Ethnicity, *n* (%)				0.809
Chinese	0544 (50.4)	455 (51.0)	089 (47.6)	
Malay	0263 (24.4)	217 (24.3)	046 (24.6)	
Indian	0136 (12.6)	110 (12.3)	026 (13.9)	
Others	0136 (12.6)	110 (12.3)	026 (13.9)	
Maternal BMI, kg/m^2^, *n* (%)				0.542
Underweight (< 18.5)	0052 (5.5)	045 (5.6)	007 (4.8)	
Normal (18.5–24.9)	0498 (52.3)	414 (51.4)	084 (57.5)	
Overweight (25.0–29.9)	0250 (26.3)	214 (26.6)	036 (24.7)	
Obese (≥30.0)	0152 (16.0)	133 (16.5)	019 (13.0)	
Number of miscarriages, *n* (%)				0.076
1	0192 (81.4)	158 (83.6)	034 (72.3)	
≥2	0044 (18.6)	031 (16.4)	013 (27.7)	
Nausea, *n* (%)				0.008
No	0591 (66.6)	480 (64.8)	111 (76.0)	
Yes	0296 (33.4)	261 (35.2)	035 (24.0)	
Pain, *n* (%)				0.154
No	0652 (61.4)	547 (62.4)	105 (56.8)	
Yes	0410 (38.6)	330 (37.6)	080 (43.2)	
PBAC score, *n* (%)				0.587
0	025 (2.4)	0023 (2.6)	2 (1.1)	
1	867 (81.6)	718 (81.8)	149 (80.5)	
2	24 (2.3)	18 (2.1)	6 (3.2)	
3	6 (0.6)	5 (0.6)	1 (0.5)	
	**All**	**Miscarriage status at 16 weeks' gestation**		***P*** **-value**
**Characteristic**	**(*****n*** = **1,080)**	**Ongoing pregnancy (*****n*** = **893)**	**Clinical factors**	
**Clinical factors**				
Smoker, *n* (%)				0.287
No	177 (93.2)	26 (86.7)	151 (94.3)	
Yes	13 (6.8)	4 (13.3)	9 (5.6)	
**Radiological factors**				
Gestation age by ultrasound, weeks (SD)	7.28 ± 1.71	7.51 ± 1.77	6.20 ± 0.77	< 0.001
Fetal heart, *n* (%)				< 0.001
No	151 (14.0)	62 (6.9)	89 (47.6)	
Yes	929 (86.0)	831 (93.1)	98 (52.4)	
**Biochemical factors**				
Serum progesterone ≥ 35 nmol/L, *n* (%)				< 0.001
No	198 (18.4)	61 (6.9)	137 (73.3)	
Yes	877 (81.6)	827 (93.1)	50 (26.7)	
Progesterone treatment given at the UOGC, *n* (%)				< 0.001
No	823 (76.2)	772 (86.5)	51 (27.3)	
Yes	257 (23.8)	121 (13.5)	136 (72.7)	

Continuous variables were analyzed via a two-sample t-test and presented as mean values with standard deviations, while categorical variables were analyzed using Chi-square test and are presented as frequencies and percentages.

BMI, body mass index; PBAC, Pictorial Bleeding Assessment Chart.

Missing data: race (*n* = 1); maternal BMI (*n* = 128); nausea (*n* = 193); pain (*n* = 18); PBAC score (*n* = 17); smoker (*n* = 890); serum progesterone ≥ 35 nmol/L (*n* = 5).

### 3.2 Univariable analysis of risk factors and multivariable prediction models for spontaneous miscarriage

The univariable analyses for each clinical, biochemical and radiological factor were presented with the corresponding AUROC values (Appendix 2). Higher maternal age, absence of nausea, an increased number of previous miscarriages, low serum progesterone levels, lower gestational age, and absence of a fetal heart on ultrasound scan significantly increased the odds of miscarriage at 16 weeks. Notably, a low serum progesterone level demonstrated strong predictive performance, with an AUROC of 0.83 (95% CI: 0.80–0.86) and a significantly increased odds ratio of 36.92 (95% CI: 24.37–55.92) for miscarriage.

Table 2 shows the multivariable prediction models for miscarriage at 16 weeks, accounting for clinical history, blood test and ultrasound scan results. Inclusion of all three types of risk factors (Model 1) yielded an AUROC score of 0.90 (95% CI: 0.87–0.93). The inclusion of only four features (maternal age, serum progesterone, absence of fetal heart and gestational age) in Model 2 yielded a similar AUROC of 0.90 (95% CI: 0.87–0.93). Considering the clinical factors alone (Model 5), the ROC analysis yields AUROC of 0.57 (95% CI: 0.53–0.62). The inclusion of either a serum progesterone test (Model 3) or a scan (Model 4) yielded model performance to AUROC of 0.85 (95% CI: 0.82–0.89) and AUROC of 0.80 (95% CI: 0.77–0.84) respectively. Model 2 in Table 3 was chosen to be the final risk-score model for miscarriage with a similar AUROC of 0.90 as model 1 and with fewer variables. Sensitivity analysis of the complete dataset shared similar results (Appendix 3).

**Table 2:** Multivariable prediction models for spontaneous miscarriage at 16 weeks in patients with threatened miscarriage.

**Characteristic**	**Model 1 (*n* = 1,080)**	**Model 2 (*n* = 1,080)**	**Model 3 (*n* = 1,080)**	**Model 4 (*n* = 1,080)**	**Model 5 (*n* = 1,080)**
	**aOR (95% CI)**	**aOR (95% CI)**	**aOR (95% CI)**	**aOR (95% CI)**	**aOR (95% CI)**
**Clinical factors**					
Maternal age (years)	1.05 (1.00–1.11)	1.05 (1.00–1.11)	1.06 (1.02–1.11)	1.02 (0.98–1.07)	1.04 (1.01–1.08)
Absence of nausea	1.34 (0.79–2.27)		1.69 (1.03–2.78)	1.33 (0.85–2.08)	1.62 (1.10–2.40)
Number of miscarriages	1.06 (0.73–1.56)		1.13 (0.79–1.61)	1.20 (0.89–1.62)	1.33 (1.03–1.73)
**Biochemical factors**					
Low serum progesterone levels (< 35 nmol/L)	26.3 (16.6–41.6)	26.3 (16.6–41.5)	38.4 (25.1–59.0)		
**Radiological factors**					
Gestational age (weeks)	0.57 (0.45–0.73)	0.57 (0.45–0.73)		0.54 (0.44–0.66)	
Absence of fetal Heart	3.91 (2.28–6.71)	4.05 (2.37–6.92)		6.31 (4.16–9.56)	
AUROC (95% CI)	0.90 (0.87–0.93)	0.90 (0.87–0.93)	0.85 (0.82–0.89)	0.80 (0.77–0.84)	0.57 (0.53–0.62)
AIC	496.1	495.2	562.8	706.0	887.5

Multivariable analyses of clinical, blood test and radiological factors for miscarriage risk at 16 weeks in a cohort of patients with threatened miscarriage. Characteristics analyzed include maternal age, absence of nausea, serum progesterone levels, gestational age, and absence of fetal heart. Adjusted odds ratios (aOR) and 95% confidence intervals (CI) were presented. The aOR is adjusted odds ratio for all other factors in the given model. The area under the receiver operating characteristic curve (AUROC) values and Akaike information criterion (AIC) for each model were presented. The 95% CI of AUROC was calculated using bootstrapping with 2,000 replications.

**Table 3:** Risk score model for spontaneous miscarriage at 16 weeks' gestation (*n* = 1,075).

	**aOR (95% CI)**	**β coefficient**	**Score**
**Maternal age (years)**			
< 40	1.00		0
≥40	5.11 (1.8–14.48)	1.63	2
**Serum progesterone levels (nmol/L)**			
< 35	28.42 (17.98–44.91)	3.35	3
≥35	1.00		0
**Gestational age (weeks)**			
< 6.36	2.86 (1.77–4.64)	1.05	1
≥6.36	1.00		0
**Fetal heart**			
No	4.53 (2.62–7.86)	1.51	2
Yes	1.00		0

Coefficient of logistic regression model, adjusted odds ratios (aOR) and 95% confidence intervals (CI) are presented. The risk score values were estimated based on the range of β coefficients in the model. Score 1: < 1.10; Score 2: 1.10–2.00; Score 3: >2.00. The model at optimal cut off point of 2.5 had sensitivity of 0.80 and specificity of 0.87. Average AUC calculated from cross validation: 0.89. The 95% CI of AUROC (0.85–0.92) was calculated using bootstrapping with 2,000 replications.

### 3.3 Risk score model for spontaneous miscarriage

**Figure 1:**
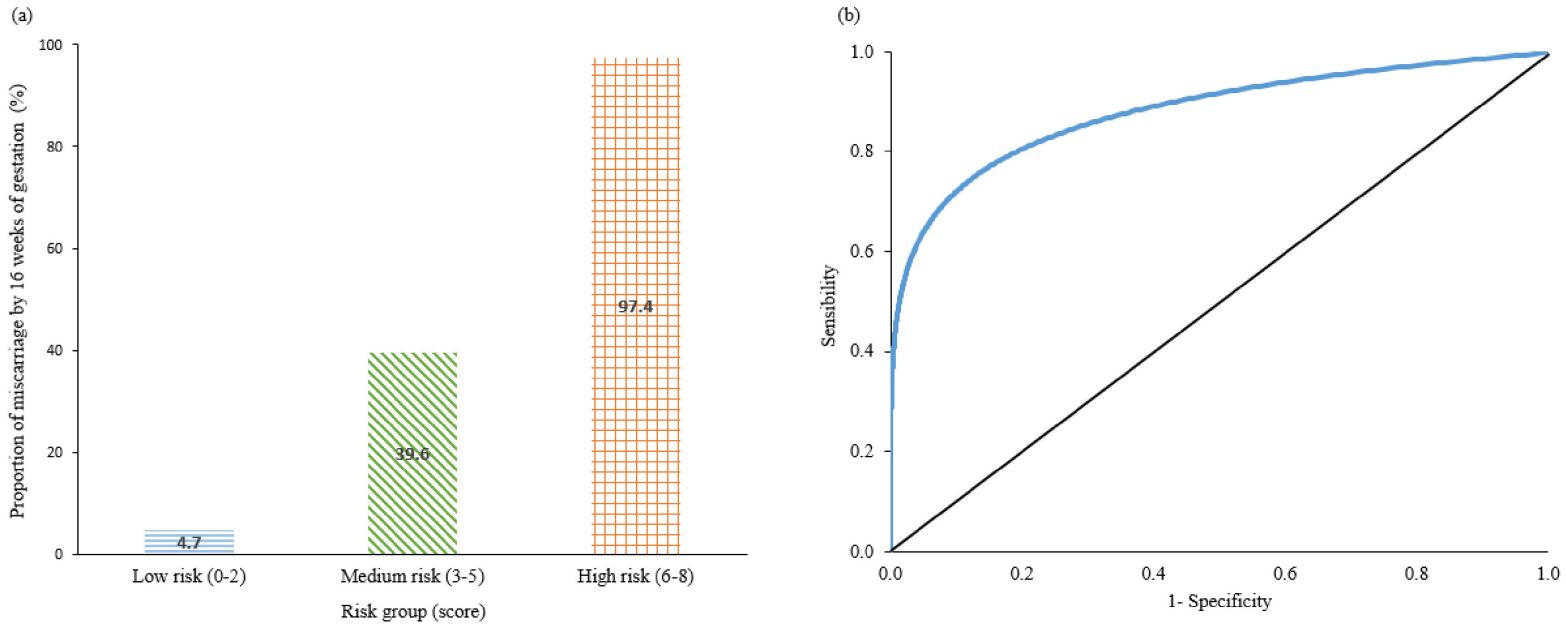
**(a)** Bar chart represent the Proportion of miscarriage by 16 weeks' of gestation in percentage in low risk (score 0–2), medium risk (3–5), and high risk (6–8) groups. **(b)** ROC performance of risk-score model. ROC, receiver-operating characteristic curve.

The risk-score model for miscarriage at 16 weeks is shown in Table 3. The cut-off of gestational age of 6.36 weeks was selected with sensitivity 0.67, specificity 0.74, positive predictive value (PPV) 31.9% and negative predictive value (NPV) 92.4%. The range of risk scores were between 0 and 8. The total risk scores of each participant were calculated using the formula: Miscarriage risk score = 2^*^(age ≥ 40 years) + 3^*^(serum progesterone level < 35 nmol/L) + (gestation age < 6.36 weeks) + 2^*^(absence of fetal heart). The proportion of women who experienced a miscarriage was 4.7% (38/811) in the low-risk group (score 0–2), 39.6% (74/187) in the medium risk group (score 3–5), and 97.4% (75/77) in the high-risk group (score 6–8) (Figure 1a). This risk assessment model demonstrated good discriminatory ability, achieving an AUROC of 0.82, with sensitivity and specificity, PPV and NPV values of 0.80, 0.84, 0.51 and 0.95, respectively, at the optimal cut-off point of 2 (Table 3, Figure 1b; Appendix 4).

## 4 Discussion

### 4.1 Main findings

Our study introduced novel risk prediction models for miscarriage among women presenting with threatened miscarriage, integrating key clinical, biochemical, and radiological parameters. Each model was developed using a unique combination of these factors, highlighting the relative importance of each in predicting miscarriage risk. The risk assessment model comprising maternal age, serum progesterone, gestational age, and fetal heart can readily be implemented in clinical settings to predict the risk of miscarriage.

### 4.2 Interpretation

Surprisingly, certain factors traditionally considered as predictors, such as maternal BMI and smoking status (current or previous), did not reach statistically significance in our models. While maternal BMI is widely recognized for its bimodal relationship with miscarriage risk (i.e. increasing risk for individuals with underweight or obesity) (29), this association was absent in our sample. Given that 16% of our participants were classified as obese, the lack of significance is not merely a result of sample representation. Instead, it may reflect characteristics unique to our study population, which consists of individuals experiencing threatened miscarriage, rather than a general pregnancy cohort. It is possible that BMI exerts a lesser effect in populations already presenting with other miscarriage risk factors which are more significant, effectively attenuating the BMI association seen in the general population. However, further research should examine the role of BMI and the broader impact of metabolic health on miscarriage risk among women with threatened miscarriage.

Consistent with prior research, serum progesterone emerged as the strongest predictor of miscarriage (13). Our final prediction model incorporated maternal age, serum progesterone level, gestational age, and absence of fetal heart on ultrasound, achieving an AUROC of 0.90. Low serum progesterone levels were substantially associated with increased odds of miscarriage. This finding aligns with existing studies that consistently associate higher progesterone levels with a reduced risk of miscarriage (30). The reported cut-off values for serum progesterone in early pregnancy may vary considerably across studies, largely due to heterogeneity in assay methodologies, gestational timing of sampling, and population characteristics. In general, while women with ongoing pregnancies exhibit a progressive rise in serum progesterone with advancing gestation, those who miscarry fail to show this physiological increase (12). This likely reflects underlying corpus luteum insufficiency and suboptimal luteal–placental transition, which impair endometrial receptivity and early placental development (31). These results underscore the potential utility of serum progesterone as a screening tool for threatened miscarriage, and progesterone supplementation may therefore restore hormonal support and stabilize the decidual–trophoblastic interface, mitigating the risk of pregnancy loss (32).

In contrast to low serum progesterone, the inclusion of the number of prior miscarriages did not significantly enhance predictive performance of our model. While existing guidelines recommend using a history of miscarriage as a triage tool (1), evidence suggests that an elevated risk may only become clinically significant with a history of three or more miscarriages (7). While the clinical relevance of miscarriage history in risk prediction should not be overlooked, it is best utilized as a supplementary factor rather than a standalone predictor. On the other hand, serum progesterone levels demonstrated greater utility as a primary triage tool, offering a more robust and actionable approach to risk stratification in clinical care.

Our findings underscore the clinical potential of serum progesterone as a primary predictive marker for miscarriage risk. While current guidelines prioritize a history of miscarriage for guiding management (15), our results suggest that serum progesterone may provide superior predictive accuracy. Incorporating a serum progesterone measurement, which is non-invasive and accessible, with other clinical and radiological features, could refine miscarriage risk assessment and improve clinical decision-making (33). The integration of serum progesterone into a multifactorial model alongside maternal age, gestational age, and the absence of fetal heart activity yielded a clinically applicable risk calculator with strong discriminatory performance (AUROC 0.90). This tool effectively stratifies patients into low-, medium-, and high-risk categories, enabling individualized counseling and timely interventions, particularly for those at higher risk. Its simplicity and robust performance make it adaptable across diverse healthcare settings. The predictive strength of this multifactorial model over single factors such as history of miscarriage supports a more comprehensive, personalized approach to risk stratification. Overall, this model's practicality and robustness have promising implications for transforming miscarriage management and guiding evidence-based clinical protocols.

Future studies should aim to validate the proposed multifactorial miscarriage risk model across diverse populations and healthcare settings. Large-scale, multi-center studies are needed to ensure the model's predictive accuracy and applicability in various demographic and clinical contexts. Randomized trials recruiting women classified as high-risk by the model could potentially demonstrate the effectiveness of early interventions in improving pregnancy outcomes. Furthermore, integrating emerging serum cytokines or urine biomarkers (13, 33) along with paternal risk factors such as age, could significantly enhance the predictive accuracy of the model.

### 4.3 Strengths and limitations

This study's strengths include its large sample size and inclusion of participants from diverse Asian ethnicities and age groups, enhancing generalizability. A wide range of miscarriage risk factors were analyzed in multiple combinations, allowing flexible model use based on resource availability, particularly valuable in low-resource settings where ultrasound availability may be limited.

Nonetheless, this study has limitations. While the prospective cohort design enables temporal associations between risk factors and outcomes, potential loss to follow-up and missing data may affect the completeness and accuracy of results, limiting generalizability. Additionally, as an observational study, it cannot establish causality due to possible confounding. Furthermore, a proportion of women with low serum progesterone received progestogen supplementation, introducing potential treatment-related confounding that may have attenuated the observed association and underestimated the true predictive strength of progesterone for miscarriage risk. Future studies with larger sample sizes and more rigorous follow-up are needed to validate and strengthen the model's predictive performance.

## 5 Conclusions

Our risk assessment model accurately predicts miscarriage by 16 weeks in women with threatened miscarriage using accessible clinical, biochemical, and radiological factors. It offers a practical tool for early identification and personalized management, with potential to improve outcomes and guide clinical care. Future studies should aim to validate this model across diverse populations and facilitate the implementation in clinical settings.

## Data Availability

The raw data supporting the conclusions of this article will be made available by the authors, without undue reservation.
