# Supplementary material for: A risk prediction model of spontaneous miscarriage in women with threatened miscarriage: a prospective cohort study
Source: Front Med (Lausanne). 2025 Nov 11;12:1669594. doi: 10.3389/fmed.2025.1669594 (PMC12644018; doi:10.3389/fmed.2025.1669594)
Supplement: Supplementary file 1 [file Supplementary_file_1.docx]

**
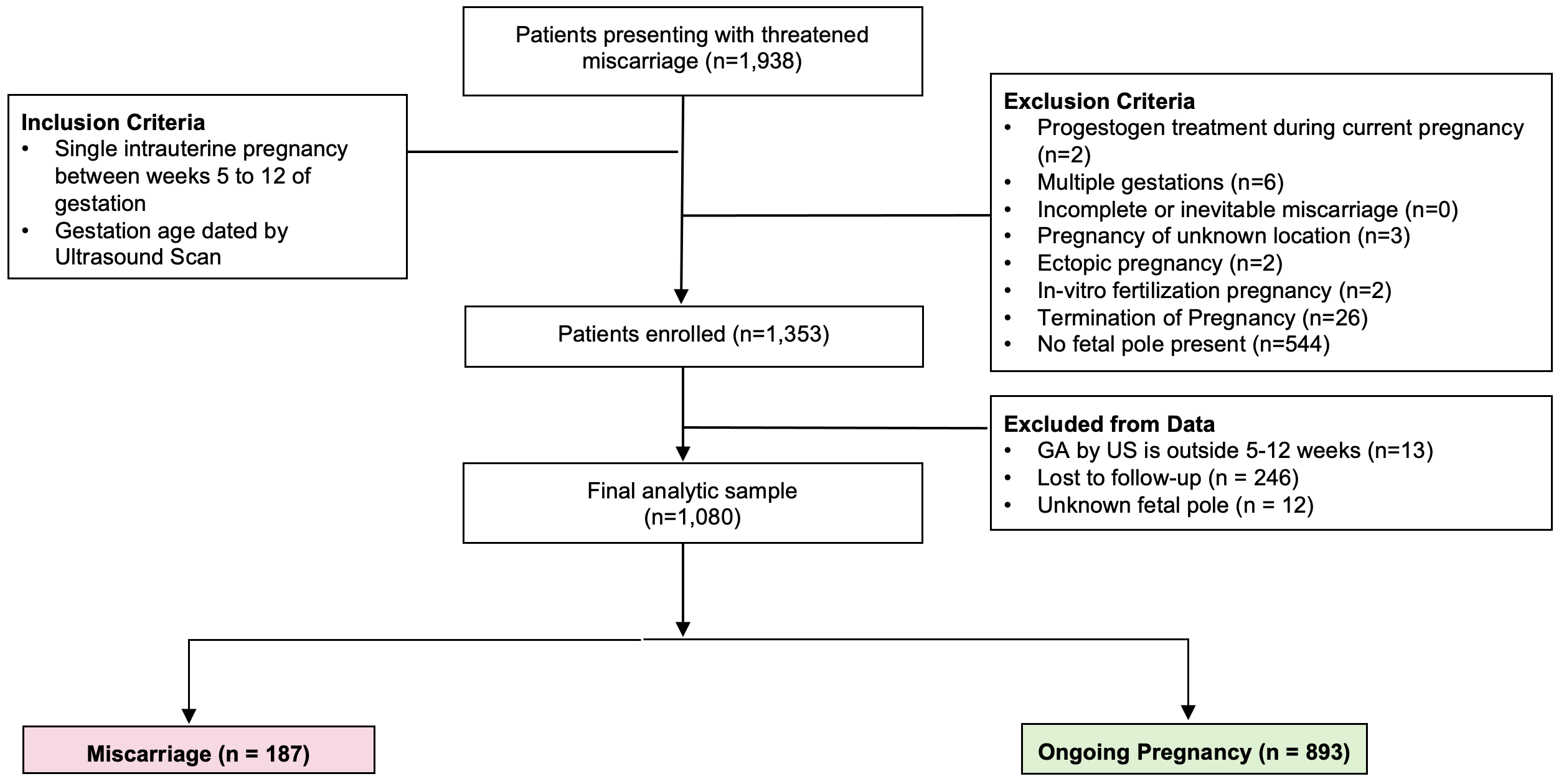
**

**Appendix 1. Flowchart of clinical outcomes of patients presenting with threatened miscarriage**

**Appendix 2. Univariable analysis of risk factors for spontaneous miscarriage at 16 weeks in patients with threatened miscarriage (n=1080)**

|  | **Factor** | **Crude OR (95% CI)** | **AUROC (95% CI)** |
| --- | --- | --- | --- |
| **Clinical Factors** | Maternal age (years) | 1.04 (1.01-1.08) | 0.55 (0.50-0.60) |
|  | Absence of Nausea | 1.59 (1.08-2.34) | 0.55 (0.51-0.59) |
|  | Number of miscarriages | 1.36 (1.05-1.76) | 0.52 (0.49-0.56) |
| **Biochemical Factors** | Low serum progesterone levels (<35nmol/L) | 36.92 (24.37-55.92) | 0.83 (0.80-0.86) |
| **Radiological Factors** | Gestational Age (weeks) | 0.41 (0.33-0.50) | 0.25 (0.22-0.29) |
|  | Absence of Fetal Heart | 12.0 (8.33-17.9) | 0.30 (0.26-0.34) |

Abbreviations: OR, Odds ratio; CI, confidence interval; AUROC, area under the receiver operating characteristic.

**Appendix 3.** **Multivariable predictive models for miscarriage at 16 weeks in patients with threatened miscarriage using the complete dataset (n=881)**

| **Characteristic** | **Model 1** | **Model 2** | **Model 3** | **Model 4** | **Model 5** |
| --- | --- | --- | --- | --- | --- |
|  | aOR   (95% CI) | aOR  (95% CI) | aOR  (95% CI) | aOR  (95% CI) | aOR  (95% CI) |
| **Clinical Factors** | | | | | |
| Maternal age (years) | 1.06 (1-1.13) | 1.06 (1-1.13) | 1.07 (1.02-1.13) | 1.04 (0.99-1.09) | 1.06 (1.02-1.1) |
| Absence of Nausea | 1.48 (0.84-2.6) |  | 1.93 (1.13-3.28) | 1.42 (0.89-2.25) | 1.78 (1.18-2.68) |
| Number of miscarriages | 1.07 (0.69-1.67) |  | 1.13 (0.76-1.7) | 1.12 (0.79-1.59) | 1.22 (0.9-1.64) |
| **Biochemical Factors** | | | | | |
| Low serum progesterone levels (<35nmol/L) | 26.76 (15.82-45.28) | 26.55 (15.74-44.79) | 39.32 (24.31-63.6) |  |  |
| **Radiological Factors** | | | | | |
| Gestational Age (weeks) | 0.59 (0.45-0.77) | 0.59 (0.45-0.76) |  | 0.58 (0.47-0.73) |  |
| Absence of Fetal Heart | 4.75 (2.6-8.67) | 4.93 (2.71-8.98) |  | 8.22 (5.14-13.14) |  |
| **AUROC (95% CI)** | **0.90 (0.87-0.93)** | **0.90 (0.87-0.93)** | **0.85 (0.81-0.89)** | **0.82 (0.78-0.86)** | **0.57 (0.52-0.63)** |
| **AIC** | **430.5** | **428.4** | **497.9** | **605.9** | **781.3** |

Multivariable analyses of clinical, blood test and radiological factors for miscarriage risk at 16 weeks in a cohort of patients with threatened miscarriage. Characteristics analysed include maternal age, absence of nausea, serum progesterone levels, gestational age, and absence of fetal heart. Adjusted odds ratios (aOR) and 95% confidence intervals (CI) are presented. The aOR is adjusted odds ratio for all other factors in the given model. The area under the receiver operating characteristic curve (AUROC) values and Akaike information criterion (AIC) for each model are also presented. The 95% CI of AUROC was calculated using bootstrapping with 2000 replications.

**Appendix 4. Sensitivity, specificity, PPV and NPV of Multivariable predictive models for miscarriage at 16 weeks in patients with threatened miscarriage at different threshold)**

| Thresholds of risk score | Sensitivity | Specificity | PPV | NPV |
| --- | --- | --- | --- | --- |
| ≥1 | 90.4% | 59.3% | 31.9% | 96.7% |
| ≥2 | 80.2% | 84.0% | 51.4% | 95.3% |
| ≥3 | 79.7% | 87.0% | 56.4% | 95.3% |
| ≥4 | 62.0% | 97.6% | 84.7% | 92.4% |
| ≥5 | 44.9% | 99.8% | 97.7% | 89.6% |
| ≥6 | 40.1% | 99.8% | 97.4% | 88.8% |
| ≥7 | 4.3% | 100.0% | 100.0% | 83.2% |
| ≥8 | 4.3% | 100.0% | 100.0% | 83.2% |

Abbreviations: PPV: Positive Predicted Value; NPV: Negative Predicted Value.
